# Glutathione S-transferases and UDP-glycosyltransferases Are Involved in Response to Aluminum Stress in Flax

**DOI:** 10.3389/fpls.2016.01920

**Published:** 2016-12-21

**Authors:** Alexey A. Dmitriev, George S. Krasnov, Tatiana A. Rozhmina, Natalya V. Kishlyan, Alexander V. Zyablitsin, Asiya F. Sadritdinova, Anastasiya V. Snezhkina, Maria S. Fedorova, Olga Y. Yurkevich, Olga V. Muravenko, Nadezhda L. Bolsheva, Anna V. Kudryavtseva, Nataliya V. Melnikova

**Affiliations:** ^1^Engelhardt Institute of Molecular Biology, Russian Academy of SciencesMoscow, Russia; ^2^All-Russian Research Institute for FlaxTorzhok, Russia

**Keywords:** *Linum usitatissimum*, flax, aluminum stress, acid soil, transcriptome sequencing, gene expression, glutathione S-transferase, UDP-glycosyltransferase

## Abstract

About 30% of the world's ice-free land area is occupied by acid soils. In soils with pH below 5, aluminum (Al) releases to the soil solution, and becomes highly toxic for plants. Therefore, breeding of varieties that are resistant to Al is needed. Flax (*Linum usitatissimum* L.) is grown worldwide for fiber and seed production. Al toxicity in acid soils is a serious problem for flax cultivation. However, very little is known about mechanisms of flax resistance to Al and the genetics of this resistance. In the present work, we sequenced 16 transcriptomes of flax cultivars resistant (Hermes and TMP1919) and sensitive (Lira and Orshanskiy) to Al, which were exposed to control conditions and aluminum treatment for 4, 12, and 24 h. In total, 44.9–63.3 million paired-end 100-nucleotide reads were generated for each sequencing library. Based on the obtained high-throughput sequencing data, genes with differential expression under aluminum exposure were revealed in flax. The majority of the top 50 up-regulated genes were involved in transmembrane transport and transporter activity in both the Al-resistant and Al-sensitive cultivars. However, genes encoding proteins with glutathione transferase and UDP-glycosyltransferase activity were in the top 50 up-regulated genes only in the flax cultivars resistant to aluminum. For qPCR analysis in extended sampling, two UDP-glycosyltransferases (UGTs), and three glutathione S-transferases (GSTs) were selected. The general trend of alterations in the expression of the examined genes was the up-regulation under Al stress, especially after 4 h of Al exposure. Moreover, in the flax cultivars resistant to aluminum, the increase in expression was more pronounced than that in the sensitive cultivars. We speculate that the defense against the Al toxicity *via* GST antioxidant activity is the probable mechanism of the response of flax plants to aluminum stress. We also suggest that UGTs could be involved in cell wall modification and protection from reactive oxygen species (ROS) in response to Al stress in *L. usitatissimum*. Thus, GSTs and UGTs, probably, play an important role in the response of flax to Al *via* detoxification of ROS and cell wall modification.

## Introduction

About 30% of the world's ice-free land area is occupied by acid soils (Von Uexküll and Mutert, 1995). Soil acidification results from acidic precipitation, deposition of acidifying gasses, or particles from the atmosphere, application of acidifying fertilizers, and mineralization of organic matter (Goulding, 2016). Moreover, anthropogenic pressure can result in further soil acidification (Guo et al., 2010; Lawrence et al., 2013). In soils with pH below 5, aluminum (Al) is solubilized to Al^3+^ ionic forms, which are released into the soil solution, and become highly toxic for plants by inhibiting the root function and growth (Kinraide, 1991; Zheng, 2010). Lands, which are preferable for plant cultivation, are already in agricultural use. Intensive soil exploitation can result in soil erosion and further decrease in cultivable areas. As human population is increasing rapidly, crop production also needs to keep pace with it (Godfray et al., 2010). Therefore, plant cultivation on unfavorable soils is necessary and breeding of varieties, which are resistant to Al and other stress factors, is needed.

The search for mechanisms involved in the response of plants to Al has revealed different strategies for adaptation, including Al avoidance and Al tolerance. One of the best-characterized mechanisms is organic acid exudation to chelate Al^3+^ and prevent its entry into the root (Yang et al., 2013). Aluminum tolerance mechanisms include detoxification of its harmful compounds, modification of cell wall properties, transport of Al to shoots, and its storage in innocuous forms, etc. (Grevenstuk and Romano, 2013; Kochian et al., 2015; Sade et al., 2016).

Diverse plant species have different strategies for Al resistance; these have been described for various plants, including wheat (Delhaize et al., 1993; Garcia-Oliveira et al., 2013; Moustaka et al., 2016), barley (Furukawa et al., 2007; Ma et al., 2016), sorghum (Magalhaes et al., 2007; Caniato et al., 2014), rice (Ma et al., 2002; Yokosho et al., 2011; Xia et al., 2013; Arenhart et al., 2014), maize (Piñeros et al., 2002; Maron et al., 2013), *Arabidopsis* (Liu et al., 2009; Mangeon et al., 2016), snap bean (Miyasaka et al., 1991), buckwheat (Zhu et al., 2015), eucalyptus (Tahara et al., 2014), and hydrangea (Negishi et al., 2013).

Flax (*Linum usitatissimum* L.) is grown worldwide for fiber and seeds and has attracted the attention of scientists (Muir and Westcott, 2003; Johnson et al., 2011; Wang et al., 2012; Melnikova et al., 2014a,b). *L. usitatissimum* has *2n* = 30 chromosomes, whereas the chromosome number in different species of the genus *Linum* varies from *2n* = 16 to *2n* = 84 (Rogers, 1982; Bolsheva et al., 2015). Nuclear DNA content of flax has been evaluated to be 352 Mb by reassociation kinetics analysis and 373 Mb by flow cytometry (Cullis, 1981; Wang et al., 2012). The *L. usitatissimum* genome has been sequenced using whole-genome shotgun sequencing, and has been predicted to contain 43,384 protein-coding genes (Wang et al., 2012). High-throughput sequencing approaches have also been applied to determine the genetic polymorphism within flax genotypes (Fu and Peterson, 2012; Kumar et al., 2012; Galindo-Gonzalez et al., 2015; Fu et al., 2016). Expression analysis allows scientists to identify genes that are expressed in particular flax tissues (Day et al., 2005; Venglat et al., 2011; Zhang and Deyholos, 2016). Moreover, the responses of flax to drought (Dash et al., 2014), salinity, and alkalinity stresses (Yu et al., 2016), nutrient imbalance (Dmitriev et al., 2016), and *Fusarium oxysporum* infection (Wojtasik et al., 2015, 2016) have been studied and stress-responsive genes identified.

Al toxicity in acid soils is a serious problem for cultivation and rich harvest of flax (Kishlyan and Rozhmina, 2010). However, very little is known about the mechanisms of resistance of flax to Al and the genetics of resistance. It has been shown that high concentration of boron affects the phenolic metabolism of flax and decreases the Al toxicity (Heidarabadi et al., 2011). In the present work, we sequenced the transcriptomes of Al-resistant and -sensitive flax cultivars grown under control or Al-treated conditions to identify genes involved in Al resistance. We also evaluated the expression of genes that potentially participate in the Al response in extended sampling using qPCR and suggested probable mechanisms for aluminum resistance in *L. usitatissimum*.

## Materials and methods

### Plant material

Flax (*L. usitatissimum*) plants of two cultivars resistant (Hermes and TMP1919) and two sensitive (Lira and Orshanskiy) to aluminum stress were used in this study. The seeds were germinated on filter paper soaked with distilled water at 21°C for 5 days. The seedlings were then transferred to Falcon tubes containing filter paper soaked in 0.5 mM CaCl_2_ solution at pH 4.5 and adapted for 24 h. Thereafter, to assess the effect of Al, the seedlings were grown for 1 day under different conditions: (1) in the presence of 0.5 mM CaCl_2_ solution at pH 4.5 for 24 h (N); (2) in the presence of 0.5 mM CaCl_2_ solution at pH 4.5 for 20 h and then in the presence of 0.5 mM CaCl_2_ solution containing 500 μM AlCl_3_ at pH 4.5 for 4 h (Al-4); (3) in the presence of 0.5 mM CaCl_2_ solution at pH 4.5 for 12 h and then in the presence of 0.5 mM CaCl_2_ solution containing 500 μM AlCl_3_ at pH 4.5 for 12 h (Al-12); (4) in the presence of 0.5 mM CaCl_2_ solution containing 500 μM AlCl_3_ at pH 4.5 for 24 h (Al-24). Root tips, 8–10 mm in length, were sampled and immediately frozen in liquid nitrogen. Plant samples were stored at −70°C. Total RNA was extracted from individual plants using an RNA MiniPrep kit (Zymo Research, USA). In total, 80 RNA samples were obtained: 5 from each of the four cultivars grown under N, Al-4, Al-12, and Al-24 conditions. The RNA quality and concentration were evaluated using Agilent 2100 Bioanalyzer (Agilent Technologies, USA) and Qubit 2.0 fluorometer (Life Technologies, USA). For further analysis, only high-quality RNA samples with RNA Integrity Number (RIN) not <8.0 were used.

### Transcriptome sequencing

The RNA samples from each cultivar grown under the same conditions were pooled in equimolar concentrations and 16 pooled RNA samples from the four cultivars under N, Al-4, Al-12, and Al-24 conditions were used for cDNA library preparation with TruSeq RNA SamplePrep (Illumina, USA). The quality of 16 libraries thus obtained was evaluated using Agilent 2100 Bioanalyzer (Agilent Technologies). Eventually, the libraries were sequenced on HiSeq2500 (Illumina) platform.

### Transcriptome assembly and differential expression analysis

Illumina reads for each cultivar were trimmed and filtered using Trimmomatic (Bolger et al., 2014) and then transferred for transcriptome assembly to Trinity, which was used with default parameters (Grabherr et al., 2011). The quality of all the four assemblies (Lira, Orshanskiy, Hermes, and TMP1919) was assessed with N50. Contigs <200 nucleotides were excluded from the further analysis. The derived transcript sequences were analyzed for the presence of ORF using TransDecoder (Haas et al., 2013). The transcripts and their predicted proteins were annotated using Trinotate (http://trinotate.github.io/). The transcripts and proteins were aligned to the UniProt database using blastx and blastp, respectively. For each transcript/protein, only the best blast hit was chosen for further analysis. The protein sequences were scanned for the presence of PFAM domains using HMMER (Punta et al., 2012; Finn et al., 2015). Based on these data, a local SQLite database was constructed and transferred to Trinotate. The mapped transcripts and proteins were annotated with Gene Ontology, KEGG, and COG entries.

The reads were mapped to the assembled transcripts and quantified using bowtie2 (Langmead and Salzberg, 2012) and rsem (Li and Dewey, 2011) at both the transcript and gene levels by two methods: (1) for each cultivar, the reads were mapped to the corresponding transcriptome; (2) the reads from all the cultivars were mapped to the Hermes transcriptome. Each method has its own disadvantages, namely: (1) the sets of assembled transcripts may significantly differ between the cultivars; (2) the cultivars may be genetically divergent, and reads can fail to align. The second method led to better results and allowed us to obtain more consensual expression data in groups of sensitive and resistant cultivars. We used bowtie2, because it is more tolerant to mismatches and indels than bowtie.

The derived read count data were analyzed using edgeR (Robinson et al., 2010). Transcripts with CPM (count per million) below 1.5 were filtered out. After normalization with TMM method, we applied approximation of the observed expression levels with two generalized linear models (GLM; values are presented for N, Al-4, Al-12, and Al-24 conditions): [0,1,2,3] and [0,1,1,1]. Approximation with the first model allows the identification of genes whose expression levels gradually change with the exposure time. The second model describes genes that are differentially expressed under any time of Al exposure. GLM [0,1,1,1] allowed us to find alterations that were specific to resistant cultivars. The results obtained with this model were used for choosing differentially expressed transcripts for further qPCR analysis. To assess the alterations of expression for each transcript in case of GLM [0,1,1,1], *fold change* values were calculated in the pool of resistant and pool of sensitive cultivars as follows:

$$\begin{array}{l} log_{2}(FC)=log_{2}(average\;CPM\;under\;Al\text{-}4,Al\text{-}12,\;and\\ \;Al\text{-}24/CPM\;under\;N) \end{array}$$

The gene set enrichment analysis with gene ontology data was performed using Goseq (http://bioconductor.org/packages/release/bioc/html/goseq.html). The analysis of the altered KEGG pathways was done with Pathview, a Bioconductor package (Luo and Brouwer, 2013).

### qPCR analysis

For qPCR analysis, 80 RNA samples of Hermes, TMP1919, Lira, and Orshanskiy grown under N, Al-4, Al-12, and Al-24 conditions were used. The differentially expressed transcripts were identified using the transcriptome sequencing data. The primers for five of such transcripts (Table 1) were designed using ProbeFinder Software (Roche, Switzerland). PCR was performed using a 7500 Real-Time PCR System (Applied Biosystems, USA) in 20-μl reaction mix containing 1X PCR mix (GenLab, Russia), 250 nM of dNTPs mix (Fermentas, Lithuania), 300 nM of forward and reverse primers, 2 U of TaqF polymerase (GenLab), 200 nM of short hydrolysis probes from Universal ProbeLibrary (Roche), and cDNA. The following amplification program was used: 95°C for 10 min, 50 cycles of 95°C for 15 s and 60°C for 60 s. Three technical replicates were performed. *ETIF3H* and *ETIF3E* were chosen as the reference genes for the qPCR data analysis (Huis et al., 2010; Melnikova et al., 2016). All the calculations were performed using the Analysis of Transcription of Genes software (Krasnov et al., 2011). For the evaluation of expression levels, $\Delta C_{t}^{eff}$ and $\Delta \Delta C_{t}^{eff}$ values were calculated (Melnikova et al., 2015, 2016).

$$\begin{array}{lll} \Delta \Delta C_{t}^{eff} & = & median{\left(\Delta C_{t}^{eff}\right)}^{stress\;conditions\;}\\ -median{\left(\Delta C_{t}^{eff}\right)}^{normal\;conditions}\\ \Delta C_{t}^{eff} & = & {\left(C_{t}^{eff}\right)}^{reference\;gene}-{\left(C_{t}^{eff}\right)}^{target\;gene}\\ C_{t}^{eff} & = & C_{t}\times log_{2}\left(1+E\right) \end{array}$$

where *Ct* is the replicate-averaged threshold cycle, and *E* is the efficiency of reaction for each pair of the primers.

**Table 1:** **Primers and probes used in the study**.

**Primer name**	**Primer sequence**	**Probe number from Roche Universal ProbeLibrary**
GST23.2-F	AAACCCATTTCCGAATCCAT	65
GST23.2-R	TGGCATCAGTGGGTAGGTTT	
GST23-F	GAGCATGATGACACACATTGAA	16
GST23-R	CGCAGGGGAATGATACTCTC	
GSTU8-F	GGTGACTAGCTCAATCCCAATG	31
GSTU8-R	CTGCAAACTTCGTCGGGTAT	
UGT71-F	GAGGTGAGAAAGAAGGTAAAGGAAA	52
UGT71-R	TGACGATCCACCTTCATTCA	
UGT74-F	CCTTCCATAACTCCCCTCAAA	22
UGT74-R	GAAGAATGAAGAAGGGATTGTGA	
ETIF3E-F	TTACTGTCGCATCCATCAGC^*^	53
ETIF3E-R	GGAGTTGCGGATGAGGTTTA^*^	
ETIF3H-F	CAGCGTGCTTGAAGTAACCA^*^	38
ETIF3H-R	AACCTCCCTCAAGCATCTCA^*^	

* *– Primer sequences are from Huis et al. (2010)*.

Mann—Whitney and Kruskal—Wallis rank-sum tests were used for the assessment of statistical significance of the revealed expression alterations. Correlation between high-throughput sequencing (CPM) and qPCR (median($\Delta C_{t}^{eff}$)) expression data was evaluated using Spearman's correlation coefficient.

## Results

### High-throughput sequencing of flax transcriptomes under Al treatment

Sixteen cDNA flax libraries (obtained from the four cultivars under N, Al-4, Al-12, and Al-24 conditions) were sequenced using HiSeq2500. In total, 44.9–63.3 million paired-end 100-nucleotide reads were generated for each library (Sequence Read Archive – SRP089959). The transcriptomes for each cultivar were assembled separately. The assembly statistics are shown in Table 2; all four cultivars demonstrated very similar statistics. About 122–126 thousand transcripts, related to 72–75 thousand “genes,” were derived for each cultivar. The annotation of transcripts was performed for Hermes, Lira, Orshanskiy, and TMP1919 cultivars. About 60% of the transcripts were successfully mapped to UniProt using blastx. For almost 70% of the 125 thousand transcripts, we found long ORFs, 47% of which were mapped to UniProt using blastp. For 46% ORFs, PFAM domains were detected. About 18 thousand transcripts passed the CPM threshold and were used for differential expression analysis.

**Table 2:** **Transcriptome assembly statistics for the examined flax cultivars**.

	**Hermes**	**TMP1919**	**Lira**	**Orshanskiy**
Genes	74,985	72,285	75,121	75,028
Transcripts	123,953	124,071	122,572	126,408
GC-content	45	44	45	44
N50	1861	1838	1871	1857
Median contig length	765	767	757	761
Average contig length	1148	1142	1150	1150
Total assembled bases, Mb	143.5	141.7	141.0	145.2
Transcripts with found ORF	85,706	84,438	86,698	85,078
Transcripts mapped to UniProt (BLASTx)	75,222	73,921	75,526	74,936
Transcripts with ORF mapped to UniProt (BLASTp)	41,321	40,712	41,408	40,631
Transcripts with ORF mapped to PFAM (HMMER)	39,677	39,027	39,327	39,422
Transcripts annotated with KEGG	38,989	38,558	38,843	38,931
Transcripts annotated with Gene Ontology (BLAST)	45,366	45,572	45,597	45,339
Transcripts annotated with Gene Ontology (PFAM)	24,488	25,256	24,924	24,901

### Identification of aluminum responsive genes on the basis of flax transcriptome sequencing

For identification of aluminum responsive genes in flax, the expression level of each transcript was estimated for each of the four studied cultivars under N, Al-4, Al-12, and Al-24 conditions, pools of resistant and sensitive cultivars under N, Al-4, Al-12, and Al-24 conditions, and pools of resistant and sensitive cultivars under N and under Al treatment (combined Al-4, Al-12, and Al-24). For further analysis, differential gene expression under Al treatment was evaluated for the pool of resistant (S1 Table) and pool of sensitive (S2 Table) cultivars using GLM [0,1,1,1]. Gene ontology analysis was performed for top 50 up- and down-regulated genes in the resistant and sensitive cultivars. The up-regulated genes were involved in transmembrane transport and transporter activity both in the resistant (S3 Table) and sensitive (S4 Table) cultivars. However, the genes encoding proteins with glutathione transferase and UDP-glycosyltransferase activity were in the top 50 up-regulated genes only in the cultivars resistant to aluminum: the up-regulation of UDP-glycosyltransferases (*log*_2_*FC* varied from 1.0 to 2.4) and glutathione S-transferases (*log*_2_*FC* = 1.1–1.2) was observed under Al stress. UDP-glycosyltransferases are involved in various biological processes, including plant stress response (Li et al., 2015). Glutathione S-transferases are anti-oxidant enzymes, which participate in Al response (Richards et al., 1998; Ezaki et al., 2000). A majority of the top 50 down-regulated genes was involved in photosynthesis and “extracellular region” in cultivars resistant to Al (S5 Table) and in the transport of different compounds in the sensitive cultivars (S6 Table). Peroxidase genes were also in the top 50 genes down-regulated under Al treatment in flax.

We also analyzed the expression of genes encoding aluminum-activated malate transporters (ALMTs), ATP-binding cassette (ABC) transporters, sensitive to proton rhizotoxicity 1 (STOP1), and aquaporins, which are known to be involved in Al responses in other plant species (Kochian et al., 2015). ALMTs were identified to be involved in Al resistance in bread wheat (Sasaki et al., 2004), sorghum (Magalhaes et al., 2007), *Arabidopsis* (Hoekenga et al., 2006; Liu et al., 2009), etc. We detected ALMT transcripts in flax and observed a decrease or retention in ALMT gene expression in Al-sensitive cultivars (*log*_2_*FC* varied from −0.78 to 0.06) and no significant alteration in the expression in the Al-resistant cultivars (*log*_2_*FC* varied from −0.28 to 0.05). Thus, we did not observe any increase in expression in flax under Al exposure for aluminum-activated malate transporters, which are involved in one of the most studied mechanisms of plant Al avoidance, i.e., root exudation of organic acid to chelate Al^3+^. ABC transporters participate in resistance to Al in *Arabidopsis* (Larsen et al., 2005) and rice (Huang et al., 2012). We observed alterations in the expression of the ABC transporter family members in all the studied cultivars, but these changes were not unidirectional: *log*_2_*FC* varied from −1.01 to 0.96 in the resistant cultivars and from −0.75 to 1.19 in the sensitive cultivars. The ABC transporter B family member 15 was in the top 50 down-regulated genes in the sensitive flax cultivars. We also identified homologs of STOP1 (transcription factor, which is involved in aluminum resistance; Fan et al., 2016) in the flax transcriptome sequencing data, but did not observe alterations in the expression under Al stress. HmPALT and HmVALT are identified in hydrangea as Al-transporting aquaporins (Negishi et al., 2012, 2013). In flax, we observed down-regulation for the most of aquaporin family members.

It is known that exposure of plants to aluminum under acid conditions results in the induction of the aluminum resistance genes and their expression is higher in the resistant genotypes than in the sensitive ones (Liu et al., 2014; Kochian et al., 2015). Therefore, the genes that were up-regulated under Al stress in the resistant flax cultivars were the most prospectively useful for further analysis.

### qPCR analysis of gene expression in flax under aluminum stress

For qPCR analysis in extended sampling, five genes with increase in their expression under Al stress were selected. These genes encode the following proteins: probable glutathione transferase GST23 (GST23.2), glutathione transferase GST23, glutathione S-transferase U8 (GSTU8), UDP-glycosyltransferase 71K2 (UGT71), and UDP-glycosyltransferase 74F1 (UGT74) (S7 Table). Five primer pairs were designed, and the expression levels of the selected transcripts (*GST23.2, GST23, GSTU8, UGT71*, and *UGT74*) were assessed in 80 RNA samples from individual flax plants of the two resistant (Hermes and TMP1919) and two sensitive (Lira and Orshanskiy) to Al cultivars, which were grown under N, Al-4, Al-12, and Al-24 conditions (five samples for each group).

**Figure 1:**
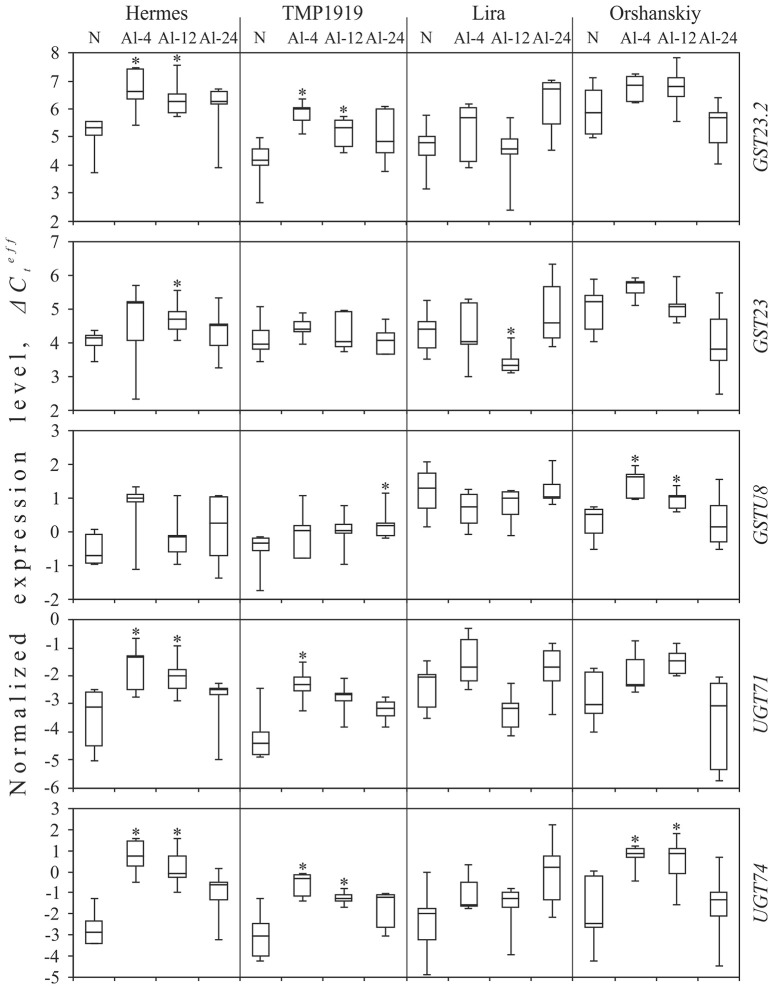
**Expression level ($\Delta C_{t}^{eff}$) of five genes under Al exposure in the resistant (Hermes and TMP1919) and sensitive (Lira and Orshanskiy) flax cultivars**. qPCR data. N, normal conditions (control); Al-4/-12/-24, aluminum exposure during 4/12/24 h. Rectangles correspond to the ranges containing 50% of the values (between the 25th and 75th percentile); the horizontal line inside the rectangle is the median value (the 50th percentiles); the bars are the maximum and minimum $\Delta C_{t}^{eff}$ values. Statistically significant (*p* < 0.05) expression changes under treatment conditions (Al-4, Al-12, or Al-24) compared to control conditions (N) are marked with asterisks.

For the *GST23.2* transcript (TR43855|c1_g1), a statistically significant increase in expression (*p* < 0.05) was observed for the resistant cultivars, Hermes, and TMP1919, under Al-4 and Al-12 conditions: $\Delta \Delta C_{t}^{eff}$ for Hermes was 1.3 under Al-4, and 1.0 under Al-12 conditions; $\Delta \Delta C_{t}^{eff}$ for TMP1919 was 1.8 under Al-4 and 1.1 under Al-12 conditions (Figure 1). For the sensitive cultivars, Lira and Orshanskiy, the tendency of alterations in the expression of *GST23.2* was similar to that in the resistant ones, but the changes were not statistically significant.

For *GST23* transcript (TR53691|c0_g1), we observed an increase in the expression under Al-4 condition for Hermes, TMP1919, and Orshanskiy cultivars, but the changes were not statistically significant. However, under the Al-12 condition, the alterations were statistically significant, but had opposite directions for Hermes ($\Delta \Delta C_{t}^{eff}$ = 0.6) and Lira ($\Delta \Delta C_{t}^{eff}$ = −1.1).

For the *GSTU8* transcript (TR41172|c0_g1), an increase in expression was observed under Al-4 condition for Hermes, TMP1919, and Orshanskiy cultivars, but it was statistically significant only for Orshanskiy ($\Delta \Delta C_{t}^{eff}$ = 1.1). Significant up-regulation was also revealed for Orshanskiy under Al-12 ($\Delta \Delta C_{t}^{eff}$ = 0.5) and for TMP1919 under Al-24 condition ($\Delta \Delta C_{t}^{eff}$ = 0.5).

*UGT71* (TR25219|c0_g1) expression was significantly increased under Al-4 conditions for both the resistant cultivars ($\Delta \Delta C_{t}^{eff}$ was 1.8 for Hermes and 2.1 for TMP1919) and under Al-12 condition only for Hermes ($\Delta \Delta C_{t}^{eff}$ = 1.2). At the same time, the increasing trend in *UGT71* expression under Al-4 was revealed for all the studied cultivars.

For the *UGT74* transcript (TR50184|c0_g1), we observed an up-regulation in the expression under all the three Al treatments for all the studied cultivars. The increase was statistically significant for Hermes, TMP1919, and Orshanskiy cultivars under Al-4 and Al-12 conditions: $\Delta \Delta C_{t}^{eff}$ was 3.6 under Al-4 and 2.8 under Al-12 for Hermes, 2.8 under Al-4 and 1.8 under Al-12 for TMP1919, and 3.3 under Al-4 and Al-12 for Orshanskiy.

Thus, the general trend of alterations in the expression in flax was the up-regulation of UGT and GST coding genes under Al stress, especially after 4 h of Al exposure. Besides, in the flax cultivars resistant to aluminum, the increase in expression was more pronounced than in the sensitive ones.

## Discussion

Aluminum toxicity in acid soils results in the decrease in the yield of flax plants. Therefore, the search for sources of *L. usitatissimum* resistance to Al is essential (Kishlyan and Rozhmina, 2010). However, for breeding of cultivars resistant to aluminum, not only the identification of resistant genotypes, but also an understanding of the genetics of resistance is required. The induction in the expression of aluminum resistance genes under Al exposure was revealed in different plant species (Liu et al., 2014). Besides, the expression of resistance genes under Al treatment was found to be higher in the resistant genotypes than in the sensitive ones (Kochian et al., 2015). Therefore, the genes with increased expression under Al treatment, especially in the resistant cultivars, are the most promising candidates for the resistance genes. For identification of the mechanisms for Al response in flax, we assessed gene expression in the cultivars resistant and sensitive to Al under control and Al treatment conditions using high-throughput sequencing and qPCR analysis. We observed the up-regulation of UDP-glycosyltransferase and glutathione S-transferase genes in flax plants under Al stress based on both high-throughput sequencing and qPCR data. The data obtained by these two methods were highly consistent: Spearman's correlation coefficient was 0.96 for *GST23.2*, 0.83 for *GST23*, 0.92 for *GSTU8*, 0.87 for *UGT71*, and 0.87 for *UGT74*. In general, in plants grown under control conditions, the mRNA levels of these genes were slightly higher in the sensitive cultivars than in the resistant ones. However, the extent of increase in expression of UGT and GST coding genes was significantly higher in the flax cultivars resistant to Al. It is worth noting that we observed the response already after 4 h of Al exposure. Thus, flax can be referred to plants that are characterized by rapid response to Al stress.

GSTs are detoxification enzymes, which catalyze the conjugation of glutathione to electrophilic compounds (Labrou et al., 2015). GSTs are involved in the response of plants to stress, including oxidative stress, and can act as glutathione-dependent peroxidases (Marrs, 1996; Dalton et al., 2009; Rahantaniaina et al., 2013). The exposure of plants to Al results in the production of reactive oxygen species (ROS) and lipid peroxidation (Gutteridge et al., 1985; Richards et al., 1998; Yamamoto et al., 2001; Jones et al., 2006). The participation of GST in the response of plants to aluminum stress was investigated, and an increase in GST expression was observed under Al stress in the resistant and sensitive to Al maize lines (Cançado et al., 2005), *Arabidopsis* (Richards et al., 1998; Ezaki et al., 2004), blueberry roots (Inostroza-Blancheteau et al., 2011), and pea roots (Panda and Matsumoto, 2010). We observed that the expression of GST gene family was up-regulated in flax plants under Al exposure, especially in the resistant cultivars. Therefore, defense against the Al toxicity *via* GST antioxidant activity, probably, is the mechanism of the response of flax plants to aluminum stress. However, for other antioxidative enzymes, which are involved in Al response, such as non-glutathione peroxidase and superoxide dismutase (Ezaki et al., 2000; Boscolo et al., 2003; Du et al., 2010), we did not observe any increase in the expression under Al stress on the basis of our high-throughput sequencing data. We suppose that GSTs play a key role in the oxidative stress defense of flax under Al treatment.

We observed that the expression of UDP-glycosyltransferase genes was also increased under Al exposure in the flax plants. UGTs are involved in the biosynthesis of secondary metabolites, hormone homeostasis, and detoxification of xenobiotics (Ross et al., 2001; Bock, 2016; Le Roy et al., 2016) and participate in the plant stress response (Chong et al., 2002; Langlois-Meurinne et al., 2005; Meissner et al., 2008; von Saint Paul et al., 2011; Li et al., 2015). The alterations in the expression of UGTs under Al exposure were observed in rice (Huang et al., 2009), buckwheat (Yokosho et al., 2014), and maize (Mattiello et al., 2014). In flax, UGTs attract special attention because of their participation in the biosynthesis of lignans—phytoestrogens with antimicrobial, antifungal, antiviral, and antioxidant activity—which have therapeutic effects against human diseases (Dixon, 2004; Pan et al., 2009; Barvkar et al., 2012; Ghose et al., 2014; Imran et al., 2015). UGTs catalyze the glucose conjugation of monolignols that is essential for normal cell wall lignification (Lin et al., 2016). Sensitive to Al rhizotoxicity 1 and 2 (STAR1 and STAR2) genes encode domains of the ABC transporter, which transport UDP-glucose that could modify the cell wall and reduce Al-toxicity (Huang et al., 2009). We suggest that UGTs could also be involved in cell wall modification in response to Al stress in flax. Besides, UGTs are implicated in the detoxification of toxins and ROS secondary metabolites (Simon et al., 2014; Krempl et al., 2016). Therefore, the protection of flax plants from ROS *via* UGTs could be another possible mechanism of *L. usitatissimum* resistance to aluminum.

Moreover, on the basis of our high-throughput sequencing data, we performed expression analysis for a number of genes, which are known to participate in Al response in different plant species, such as ALMTs, ABC transporters, STOP1, and aquaporins (Liu et al., 2014; Kochian et al., 2015). We did not observe any significant up-regulation of these genes under Al stress in flax. Probably, the enzymes encoded by these genes do not play key roles in flax resistance to Al. In *L. usitatissimum*, the detoxification of ROS and cell wall modification *via* GSTs and UGTs could be the key mechanisms for overcoming Al toxicity.

## Conclusion

We identified genes with differential expression under Al exposure in flax plants using high-throughput sequencing and qPCR analysis. We observed increase in the expression of glutathione S-transferase and UDP-glycosyltransferase genes under Al stress. Moreover, the up-regulation of these genes was more pronounced in flax cultivars resistant to Al. However, we did not notice any increase in the expression of aluminum-activated malate transporters, which are involved in one of the most studied mechanisms of plant Al avoidance—the root exudation of organic acid to chelate Al^3+^. We speculate that GSTs and UGTs are involved in the response of flax to Al stress and suggest that the probable mechanisms for the resistance of flax to aluminum are detoxification of ROS and cell wall modification *via* GSTs and UGTs.

## Author contributions

AD, TR, NB, and NM conceived and designed the work; AD, TR, NK, AZ, AFS, AVS, MF, OY, NB, and NM performed the experiments; AD, GK, AZ, OM, AK, and NM analyzed the data; AD, GK, and NM drafted the work. All the authors revised the work critically for important intellectual content, approved the version to be published, and agreed to be accountable for all aspects of the work in ensuring that questions related to the accuracy or integrity of any part of the work are appropriately investigated and resolved.

## Funding

This work was financially supported by the Russian Science Foundation, grant 16-16-00114.

### Conflict of interest statement

The authors declare that the research was conducted in the absence of any commercial or financial relationships that could be construed as a potential conflict of interest.
