# Supplementary material for: Glutathione S-transferases and UDP-glycosyltransferases Are Involved in Response to Aluminum Stress in Flax
Source: Front Plant Sci. 2016 Dec 21;7:1920. doi: 10.3389/fpls.2016.01920 (PMC5174120; doi:10.3389/fpls.2016.01920)
Supplement: Supplementary file 7 [file Table7.DOCX]

**S7 Table. Sequences of transcripts that were used in the qPCR analysis of flax plants under Al exposure.**

TR43855|c1_g1 ***GST23.2***

GCACTCCCATCACTACTATAAATATCCCTGAAGCCATTGTTTCCCAAAACCATAAACAATATCACAAAACAAATCTCAAAACCCAGAGACAATTAAAATACGTACAAATGGCAGACATCAAAGTGTTAGGAACGTGGCCTAGTCCATTCAGCTACAGAGTCATCTGGGCTTTGAAGCTGAAAGGAATAGACTACGAGTACATCCCACAAGACCTCTCCAACAAAAGCTCACTCCTCCTCCAATCCAACCCAATCCACAAGAAAATCCCAGTCCTAATCCACCAAGGAAAACCCATTTCCGAATCCATGATCATCCTCCAGTACTTGGACGAGTCATTTCCCGAAACCTACCCACTGATGCCAACCGATCCTTACCAAAAAGCCATGGCTAGATTCTGGATTAAATTTGCAGAGGACAAGCTTTCCTCTGTTTTTAAAATTTTCAGGACTTCAGGGGACGAGCAAGCAAAGGCGGTCAAGGAGACATTGGAGACTCTGACTATTCTGGAAGAACATGCTTTTGGGAGGTTGGAGAATAACAAGTTATTTGGCGGGGATAGGGTTAATATGGTTGATCTTGCATACTGCATATTGGGCTATTGGTTCGATGCAGTTGAGGATTGTACTGGAGTTGAAGTTCTGGTTGATCCTGTGAAGTTCCCGCGTATTCATGGTTGGATTGCTAGGTTTAATGACGCTCCTGTCATCAAAGATAATCTCCCTGATCGTGAAGAGATGTTTGTTAACTTCAAACGTCGTAGGGGAATTATACTTTCAGCTGCTGCTTCGTCCAACTGATAATTTAAAATGTGTGTCTTGTTTGTTGACCTTAGTCAATGCTTTTCAATAATAAATGTTTGTGCTTGTAAACTGTTTCAGGTTGTTTTGTTTGTACTTTGTTCTCATATTAATAAATAATAATCCTGAGAGTTTCTGTTTTCTTAAAAAAAAAAAAA

TR53691|c0_g1 ***GST23***

ATTTAGTGGATCCATGGGTTTAAATATACTTACCATATTGAATATATAGCAAACTTGAAATTTAAGAAATAGAATATGAACGAGATAAGATTGATTATTCAGAAGTTGTAAGAAAAAAGAGAACAAGAAAGCAGAGAAACTCTCTGGATTATTATTAAATAGAAGAACAAGTGCAACAAAAGCAACCTCAAACAGTTCACAACCACAAATACTTATTACTGACGTCGAAAGAACATAAGATGAGCATGATGACACACATTGAATTTTCAGTTGGAGGCAGCAGAGAGTATCATTCCCCTGCGACGTTTGAAGTAAGCAAACAACTCTCCACGTTCAGGAAGATTGTCTCTAATAACAGAAGCATCATTAAACCTACCAATCCAGCCATGGAAACGAGGGAACTTGAGAGGATCAAACACTTTTACTCCTGTACAGTCCTCAATAGCATCCAACCAATAACCCATCACCCCATAAGCAAGATCGACAAGGTTTACCATTTTATCACCTCCGAAATAATTAGTTCCTAACGGTCCAAAGGCATGTTCTTCCAGCACTTTCAATACCTCCAACGTCTCCCTGACTGCCTTCTCCTGCTCTTCTCCTGAAGTCCTGAATATCATGAAGGCAGATGAAAGCTTCTCCTCTGCAAATTTGATCCAGAATCTAGCCATGGCCTTCTCGTAAGGATCAGTTGGCATCAACGGGTAGGTCTCAGGGAATGACTCGTCCAAGTACTCGAGGATAATCATGGATTCGGAAATGTGTTTTCCTCCATGGATTAGGACTGGGATTTTCTTGTGGACAGGGTTGGATTGGAGGAGGAGTGAGCTTTTTTTGGAGAGGTCTTGTGGGATGTACTCATAGTCTATA

TR41172|c0_g1 ***GSTU8***

TTTTTTTTTATGAAAAACACACACAATGAACCAGTAGTATAAATAAAACTATTGGAGACAAAGAACATGATTATAAATTTGAAATGGCTTTAAAATTGACAGATAACCTGAACCGTGAAATTCAAAAACACATCAGACAAAAACTTCAATATAAAAATCTTACATAAAATTTCGTTATTTTAGAATCGAGAGCGACCCTTCATTAGTCTAGGTTTGACGTTTACTGTTTACCCACCCCTAATTCCGCGGCATGTAGGAACTTTGTTCGCAATCACAGAACATACTTCCAAGCAGGGGCGTTAGTAAGGCGAGGTATCAACGCGGCCAAGAGTTCATCTCTGGGAGGGAGAGTCTCCTTCACCTTGCTATGCCCAGCGAAGTTATCAATCCACTTGCAGAGCTCCGGAAACCTTTCAATGGTGACTAGCTCAATCCCAATGGCTTCTTCCACCACTCCCAGCCAATACCCGACGAAGTTTGCAGTTATGTCGACTATCCCGATCTCCTCGCCTCCGAAAAACGGGTTCCCCTTGAGCTGTTCTTCGAGTGTTTTCAGGTTCTGCAGCGCCTTGTGTGTCAGCTTCTCTTTCTCCTCATTGTCTTTGCAATACGCTATTTTCCACACTTCCCCGAAACACTTCTCTTGGGCAAAAGTAGCCCAAAACCGAGCCTTAGCCTTGTCGTGGAGACCAAGTGGGAATATGGGTTTGCCGGGCCATTCTTCATCGATGTACTCCAGGATAACGGCAGACTCAGAAATTGTTCTGCCGTTGTGAATAAGAACAGGGACTGTCCGGTTAACTGGATTCGACTCGAGGAGCAACGGACTCTTATCGCCGCCACTGAAGAAATCGTTCTCCAAGTAGTCGTAATCCACTCCTTTCAACTTCAGGGCGAGCTCCACCCTTTTGCTGTACGGGCTCGCCCAGAATCCCAGCACCTTCACAGTAGTTTCCATTTTTGGGTGTGAAATCACTGAGGAGTTTGGGGGATCGGATGAGAGTTATAAATAATAAACCAGAG

TR25219|c0_g1 ***UGT71***

GCCGAACCGACTCCATTTATATGGGAATATTCCACAAATATATGCCTGATCAATTTACATTCGTCGTTGAATACTCAACAGTGATTTCAAACTTTTTTTTTACAAGTTTTAAACAATTTTTTTTCATCCGTCGGAGATTCTAAGTTACATAACAAAAAAGTTCCAAGAAAAAAAACACTACATGATAAAGAAAGATTCCGCCGGCGACTCTGTTTTCCTTATAAAATTACAATCAATCCACCAGCAACTCTGCACTACGAACCATCAAAGCTTTTCAGCCAAAATTCAAAGCCCATCTCTTTAATCATGATGACTAAACCAGAGCTAGTAATGGTCCCAATTCCGGCGATCGGCCATCTCCCGCCGGTGGTCGAATTCTCCAAGCACATCACCGCTCGCAACAGTCAACTCTCCGTCACAATCGTACTCATCCGAACTCCCTTCTCTCCGGAGGTCGACTCATTTTCCGATCGGCTAGCCGAATCCTGCAAAGACTGTAAAGCAATCAATTTCATCAGAATCTCCGAACCCAAGTTCCCCCCGATCGATTCCTACAGTTCAGTACACTCTTTCTTCCCGAAATTTCTGGATTCCCAGACGGACGCTGTGAAACAGGCTCTGGCAGCTCGGTTCTCTGGCGATTCCTCTGTTTCACTGGCCGGGATTGTGGTGGATATCCTGACGACGGCGATGGTTGATTTGGGGAAGGAACTTGGTGTTCCTTCTTACCTTTTCTTCCCTTCTTGTGCAGCGATCCTGGGTCACTTAGTCCATTTGCCGGCGATGGGATACTCGCCGGAGGTAGCCGTCGGTGATCCCGATGGCGAGGTGGTTTACCCCAGCTTTGAGCATCCAATGCCTAATCGGATCCTGCCGGCGATTGTTCTCGACGGACAAGGCTACCAGGAGTTGATGGGTCACACGAGGAAGTACAATGAGGTCGATGGAATCGTTGTGAATTCGTATGTCGGGCTCGAGTCTCGAGCTATTAATATCTTGAACGGAAAAGTTGATGGAGTTTTCCGGATTGGAGGGAAGTCATTCCCTCCTGTTTTTCCAGTCGGACCGGTTCTCAATCTGAAAGGGCATGCCACGTTAGGAAACACCAAATCGTTGAGCGAAAAAGCGATGACGTGGCTAGACGACCAACCTCCACAATCGGTCGTGTTCATGTGCTTCGGAAGTCTAGGAAGCTTCACCGACGCCCAACTCGGGGAAGTCGCGGCCGGGCTAGAGCGAGCACGACATGTGCGATTTTTGTGGGTAATGCGCAAGATATCATCCGGAGATTCAAAATGGACGCCTAACGATTGCGAAGACTACTCTCCCTCCTCGCCCGCTCTAAACGCCTTGGGCGAAGGATTTCTTGAGCGGACGAGAGGGAGAGTAATGGTGTGCGGGTGGCTCCCTCAGGCAGCTATACTAGCCCACAAGGCCATTGGAGGGTTCATGTCACACTGCGGTTGGAACTCTATACTTGAGAGCCTCTGGCACGGAGTGCCCATGTTAGCATGGCCGATGTATGCAGAGCAACAGATGAATGCATTCTACATGACAACAGAGTTAGGATTAGCGGTCGAGCTGAGGGCAGACTACCGAATATGGAAGAGCGATGAGGATGGAGAAATGGTGGTGAAGGGAGATGAGATTGCTAGGAAGATTGAGATGGTGATGGACAAGCATAGTGAGGTGAGAAAGAAGGTAAAGGAAATGAGTGAGCTTGGGAGGAGGGCTTTGAATGAAGGTGGATCGTCATTTGATGGCATTGATGGATTCATGGATTTGGTATTGAAGAACAAGCCAATGGTTTGAGTACTATCCATAATTTGGATTGGTGAAGCCAATGGATTTTTTTTAAAAGCAATTTTTATTTTTTTTAATTGTTTGATGAATTCTCGTGGCATCTTTACATAAATAAAAATCGGTTCGATAAAGATAACCCTCTATTTTACCTCAAACTCAATTGTGTGCCTTGATAAATTCATAACTCG

TR50184|c0_g1 ***UGT74***

ACCTCCCCCCTTCCCCCGCCGCTTCTTTCATCATCTTCCTCCACTTGTCCGCATTCCTCTTCATCTCTTTCCCTTCTTCCCCTTCCATAACTCCCCTCAAACACTTCTCCACCATTTCCCTCTTCACAATCCCTTCTTCATTCTTCTGACACCTCACTCCCATCTTCCAAACGTCCTCTATATACTTTGCATTGGTCGCCTGGTCCGTCCACTGAGGCATCGCCACCATGGGAACTCCCAAGCTCAACCCCTCAAGGGTCGAATTCCATCCACAGTGGGTCACGAAGCAGCCCACCGCCTCGTGTTCCAGCACCTCCAGCTGCGGGCACCACGACACAATCAACCCTTTCCCGTCGATGTCAACGTCGGCGGCGAAATTGGGAGGTAGCTTGGCCACTTCCGACGCCCTTACTACCCATAAGAAGTAGTGGTTGCTGTTCTTCAAGCCGTGCGCTATCTCCTCCATTTGCTCTGCTCCAAGGCTTGCCAGGCTGCCGAATGATACATAAATTACAGAGCCTTTCGGCTTGTTGTTGTTCAGCCAATTAATGCAGGCTTCGTTATTCGGCTTGAAGATGCTGAACCCGTAATCTCTATCGTCTTGTAATTGCTTGTCCAAGTACATCGAAGGAATTGTGGGGCCCACTGTTCTCAGTGGCCAGATCGTCGATAGCCAATCCGCCGTCTCGTTCTCGAGATCGTAGACGGTGTTACAGAGGATACAGTCAGCTTTATGAATGTTGGAGAATTGGGAGATAATCATATCAAAGGCGGCGGGGTAAGAGCCGTAATCGGAGATGAAAGAAGGCATGTCATCAGCTTCCAAAGGAGCCGGCAAACCAGGGATGATCAAACTCTGCGGCTGATCAGTAACTGGAAGCTTAAGCGAGCCTTTATAGACGTGGTAGTAGATGGCGTCGACGGCACAAGACTGAGTGAAAAACGGCGCCGCAAACAATCCAAGCTCCTTGGCTACATCCAGACACCACGGAAGGAAAGGGTCGTAGATAATGCAATGGGCAGGGTACTCGGTTTTACTCAGCTTTTGAATCAGTTCGGATAGAGTCTTTGAGCCTTCTTTCTGGAAACTTTCCAAATAGGCCTGGGTGCTTTCGGCGGCAGCATGACCGCCGTCGTCGTAGCCGTCGGAGATGGTATCTAGGTTGATCGTGGAGGTGGAGGAAGTGGAGGAGATAGTTTTGGAGATGAAACGAGTGTTGACGAGAGTGATACGGATGGATTTGGAGAGGAGGCGCTTTGAGAATTGGAGCATCGGGTTGATGTGGCCTTGTGCTGGATATGGTATCACTAGGCAGTGAGGCTGTTGTTGCTTCTTCGCCGCCATTTTTTCTGAGCTGTCG
